# Clinical and Pathological Characteristics and Prognosis of Diabetic Nephropathy Patients With Tertiary Lymphoid Structures

**DOI:** 10.1155/jdr/6916580

**Published:** 2025-08-13

**Authors:** Yongjie Zhuo, Mengjie Weng, Jiaqun Lin, Xiaoting Wu, Kun Nie, Liyan Yang, Jiong Cui, Jianxin Wan

**Affiliations:** ^1^Department of Nephrology, Blood Purification Research Center, The First Affiliated Hospital of Fujian Medical University, Fuzhou, China; ^2^Fujian Clinical Research Center for Metabolic Chronic Kidney Disease, The First Affiliated Hospital of Fujian Medical University, Fuzhou, China; ^3^Department of Nephrology, National Regional Medical Center, Binhai Campus of the First Affiliated Hospital, Fujian Medical University, Fuzhou, China

**Keywords:** diabetic nephropathy, inflammation, predictive model, risk factors, tertiary lymphoid structures

## Abstract

**Purpose:** Chronic inflammation plays a key role in diabetic nephropathy (DN), yet the impact of tertiary lymphoid structures (TLSs) on disease progression remains poorly understood. This study explored the relationship between TLS maturity and renal outcomes in patients with DN.

**Methods:** This study included 117 biopsy-confirmed DN patients from the First Affiliated Hospital of Fujian Medical University. Patients were grouped based on TLS maturity, as determined by immunohistochemical staining. Clinical, laboratory, and pathological data were gathered, and Cox regression models were applied to assess renal outcome risk factors. Kaplan–Meier curves were constructed to compare renal survival among groups. A web-based model integrating TLS maturity, urinary albumin-to-creatinine ratio (UACR), and renal pathology classification was developed.

**Results:** Mature TLSs were associated with worse renal prognosis, with greater disease progression in the TLS+ group (75.4% vs. 53.3%, *p* = 0.006). Multivariate Cox regression analysis identified TLS maturity (HR = 1.819, 95% CI: 1.144–2.893, *p* = 0.011), DN glomerular classification (HR = 1.511, 95% CI: 1.057–2.160, *p* = 0.024), and UACR (HR = 1.121, 95% CI: 1.038–1.210, *p* = 0.004) as independent risk factors. The dynamic nomogram demonstrated strong predictive performance.

**Conclusions:** TLS maturity independently predicts renal function decline in DN and may support personalized risk assessment through a web-based model.

## 1. Introduction

Over the past few decades, the prevalence of diabetic nephropathy (DN) has increased significantly [1]. Data from the US Renal Data System highlight DN as the primary contributor to end-stage renal disease (ESRD) [2]. Despite advancements in diabetes treatments, current DN therapies primarily slow the decline in estimated glomerular filtration rate (eGFR), while substantial cardiovascular risks persist [3]. As progressive renal function loss often leads to kidney transplantation or renal replacement therapy, early disease detection and risk assessment are crucial.

Currently, the gold standard for diagnosing DN is through renal histology. The Renal Pathology Society (RPS) introduced a DN classification framework in 2010, categorizing glomerular, interstitial, and vascular lesions into distinct classes [4]. Research has confirmed that the RPS classification correlates with DN outcomes, as higher-grade lesions are associated with increased ESRD risk [5, 6]. However, the RPS classification does not include a specific evaluation of renal inflammation.

The precise role of chronic inflammation in renal injury and repair remains poorly understood. Interactions between renal and immune cells can initiate a vicious cycle, causing irreversible tissue damage and eventual kidney failure [7]. Tertiary lymphoid structures (TLSs), also known as tertiary lymphoid tissues or organs, are ectopic lymphoid formations that develop within nonlymphoid sites [8]. TLSs consist of T cells, B cells, follicular dendritic cells (FDCs), reticular cells, high endothelial venules, and lymphatic vessels. The developmental stages of TLS reflect the severity of local injury and inflammation, with severely damaged kidneys being capable of forming fully mature TLS with germinal center-like structures [9]. Mature TLSs harbor follicular helper T (TFH) cells that directly induce the expression of extracellular matrix proteins (fibronectin and collagen I) through IL-21-mediated activation of the NF-*κ*B pathway, thereby promoting renal interstitial fibrosis [10]. Single-cell RNA sequencing has also demonstrated that immune cell infiltration increases seven- to eightfold in DN, including monocytes, T cells, B cells, and plasma cells, which form complex interaction networks within TLSs [11]. Furthermore, spatial transcriptomics reveals that these inflammatory cells preferentially localize to fibrotic regions of DN kidneys, suggesting their interactions play a contributory role in the progression of renal fibrosis [12]. In various forms of CKD, including IgA nephropathy [13–15], IgG4-related kidney disease [16], lupus nephritis [17], interstitial nephritis [18, 19], ANCA-associated vasculitis [20, 21], membranous nephropathy [22], and renal allografts [23–29], TLS formation can occur in the kidneys. Evidence from clinical studies links TLS formation in these conditions to worse renal outcomes. TLS formation occurs within chronic inflammatory environments, and their developmental and maturation stages reflect the severity of local inflammation. Beyond histological assessment of local inflammation, several emerging inflammatory biomarkers in hematological examinations, including neutrophil-to-lymphocyte ratio (NLR), platelet-to-lymphocyte ratio (PLR), monocyte-to-lymphocyte ratio (MLR), systemic immune-inflammation index (SII), systemic inflammation response index (SIRI), and red cell distribution width-to-albumin ratio (RA), can reflect the overall inflammatory status of the body [30]. These markers are readily available in routine clinical practice, and studies have demonstrated their association with prognosis in DN patients [31, 32]. However, the relationship between these systemic inflammatory indicators and TLS, which represent local renal inflammation, as well as their relative predictive value for DN prognosis, remains to be explored. Therefore, this study included 117 DN patients confirmed by kidney biopsy to investigate the relationship between TLS maturity and patient prognosis, aiming to construct a more accurate prognostic assessment model.

## 2. Methods

### 2.1. Patients

**Figure 1:**
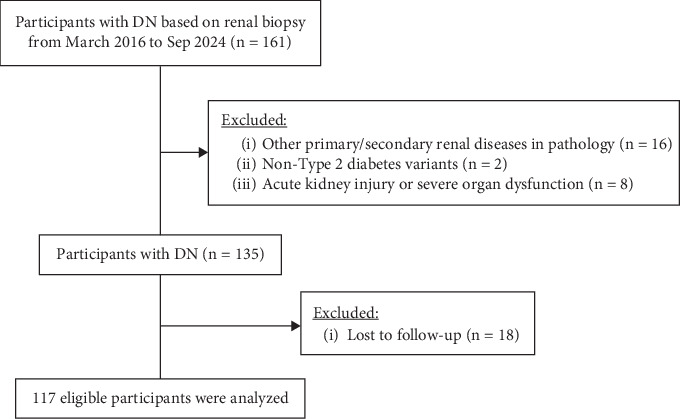
The study flowchart of the enrollment of DN patients.

This study examined the relationship between TLS and functional progression in DN patients diagnosed by renal biopsy between March 2016 and September 2024 at the First Affiliated Hospital of Fujian Medical University. The study was approved by the Institutional Review Board of Fujian Medical University, and all participants provided informed consent. Exclusion criteria included (1) age under 18 years; (2) Type 1 diabetes or steroid-induced diabetes rather than Type 2 diabetes; (3) concomitant kidney diseases, such as IgA nephropathy, lupus nephritis, or tumor-associated nephropathy; (4) reversible acute kidney injury (AKI) during follow-up; (5) follow-up of less than 6 months; and (6) incomplete data (Figure 1). Ultimately, 117 patients were included.

### 2.2. Clinical and Renal Pathological Characteristics

Medical history and clinical data at the time of renal biopsy were retrieved from the electronic medical record system, encompassing general clinical details, laboratory tests, and pathological findings. Clinical details comprised sex, age, body mass index (BMI), history of diabetes, hypertension, gout, cerebrovascular disease, smoking habits, and use of ACE inhibitors (ACEI/ARB) or lipid-lowering medications. Laboratory parameters included hemoglobin, glycated hemoglobin, total bilirubin, serum albumin, uric acid, blood urea nitrogen, creatinine, eGFR, total cholesterol, triglycerides, HDL-C, LDL-C, calcium, phosphorus, potassium, fibrinogen, complement C3 and C4, urinary protein, and UACR. Urinary protein levels, determined via dipstick test, were classified as negative (−) or graded as 1+ (30 mg/dL), 2+ (100 mg/dL), 3+ (300 mg/dL), and 4+ (≥ 1000 mg/dL). In clinical practice, renal function progression is typically defined as an eGFR decline > 25% within 1 year or an annual decline rate exceeding 5 mL/min/1.73 m^2^, a criterion that incorporates relevant recommendations from both the KDIGO 2012 guidelines [33] and NICE guidelines [34].

Renal biopsy samples underwent examination via light microscopy, immunofluorescence, and electron microscopy, following standard protocols of the First Affiliated Hospital of Fujian Medical University, and were reviewed by expert pathologists. HE, PAS, Masson's trichrome, and PASM staining were conducted to evaluate mesangial proliferation, glomerulosclerosis, interstitial inflammation, tubulointerstitial fibrosis, tubular atrophy, and arteriolar hyalinosis. Immunohistochemistry was used to examine Kimmelstiel–Wilson (K-W) nodules and deposition of CD20, CD3, CD4, CD21, and CD68 in renal tissue. The degree of renal interstitial lesions was determined based on the percentage of renal tissue area affected by interstitial fibrosis and tubular atrophy, graded as 0 (no lesions), 1 (lesions < 25%), and 2 (lesions ≥ 25%). Renal interstitial inflammation was quantified by evaluating the percentage of lymphocyte and monocyte infiltration in the renal interstitium using HE staining.

**Figure 2:**
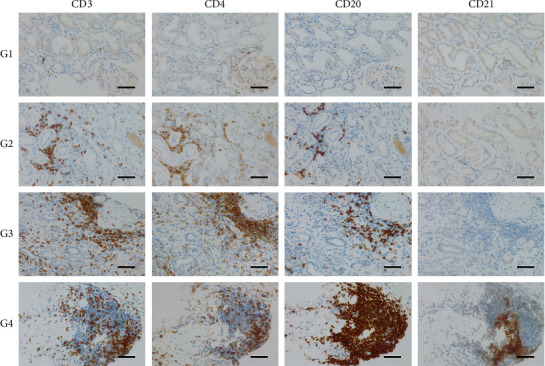
Classification of TLS maturation in renal interstitium based on immunohistochemical staining. G1: scattered CD20^+^ B cells, CD3^+^, and CD4^+^ T cells, without zonal segregation or CD21^+^ FDCs (immature TLS). G2: clustered CD20^+^ B cells and CD3^+^/CD4^+^ T cells, lacking zonal segregation and CD21^+^ FDCs. G3: clustered CD20^+^ B cells and CD3^+^/CD4^+^ T cells with defined zonal segregation but no CD21^+^ FDCs (near-mature TLS). G4: fully mature TLS with clustered CD20^+^ B cells, CD3^+^/CD4^+^ T cells, distinct zonal segregation, and CD21^+^ FDCs. TLS− (G1 and G2) represents immaturity, while TLS+ (G3 and G4) indicates functional maturation.

Morphological assessment methods for TLS have been applied in studies of various kidney diseases, including IgA nephropathy [13], lupus nephritis [17], ANCA-associated glomerulonephritis [21], renal transplantation [25], and renal clear cell carcinoma [35]. The grading criteria used in this study were based on these assessment methods, with appropriate modifications for the pathological characteristics of DN. The maturity of interstitial TLS was graded based on immunohistochemical staining, resulting in four levels. Grade 1 (G1) TLS presented as simple structures with scattered CD20^+^ B cells, accompanied by CD3^+^ and CD4^+^ T cells, lacking distinct compartmentalization or CD21^+^ FDCs, indicating an immature state. In Grade 2 (G2) TLS, clusters of CD20^+^ B cells and CD3^+^/CD4^+^ T cells began to form but without clear compartmentalization or CD21^+^ FDCs, suggesting limited maturity. Grade 3 (G3) TLS showed clusters of CD20^+^ B cells and CD3^+^/CD4^+^ T cells with distinct compartmentalization but still without CD21^+^ FDCs, suggesting a maturing TLS. Grade 4 (G4) TLS was fully mature, characterized by clustered CD20^+^ B cells and CD3^+^/CD4^+^ T cells with distinct compartmentalization and CD21^+^ FDCs. G1 and G2 were defined as TLS−, indicating an immature state, while G3 and G4 were defined as TLS+, indicating a mature state (Figure 2). All TLS assessments were independently performed by two senior pathologists with more than 10 years of experience in renal pathological diagnosis. Throughout the evaluation process, both pathologists remained blinded to patients' clinical data, laboratory results, and prognostic information. Each pathologist independently assessed all 117 patients using uniform TLS grading criteria, and their results were subsequently compared. For cases with discrepancies, the two pathologists conducted thorough discussions through joint microscopic observation and reference to the grading criteria until consensus was reached. All final TLS grades represented results agreed upon by both pathologists.

In addition to histological assessment of inflammation, this study also collected patients' complete blood count results and calculated multiple emerging systemic inflammatory biomarkers, including NLR, PLR, MLR, SII (platelet count × neutrophil count/lymphocyte count), SIRI (neutrophil count × monocyte count/lymphocyte count), and RA. All these indicators were derived from routine hematological examinations performed prior to renal biopsy.

### 2.3. Statistical Analysis

All analyses were conducted using R Version 4.5.0. Normally distributed continuous data were presented as mean ± standard deviation ($\overset{®}{x}\pm s$), while nonnormally distributed data were shown as median [M (P25, P75)]. Categorical data were expressed as frequency and percentage. Between-group comparisons used *t*-tests for normally distributed variables, while rank-sum tests were applied to nonnormally distributed and ordinal variables. Binary and unordered categorical variables were analyzed using chi-square or Fisher's exact tests. Kaplan–Meier curves assessed cumulative renal survival, with differences evaluated via the log-rank test for statistical significance. Cox proportional hazards regression was performed to estimate hazard ratios (HRs) and 95% confidence intervals (CIs) for renal outcome risk factors in DN. The proportional hazard assumption was tested to ensure the Cox model's validity. Univariate Cox regression analysis was performed to evaluate the relationship between each variable and renal outcomes. Combining key variables identified from univariate analysis with clinically important indicators for DN (glycated hemoglobin, serum creatinine, UACR, etc.) and systemic inflammatory biomarkers, LASSO-Cox regression with 10-fold cross-validation was employed for variable selection, followed by multivariate Cox regression analysis of the selected variables. These factors were further used to construct a predictive model for the derivation cohort. Discriminative ability was evaluated using ROC analysis, followed by the construction of a nomogram. A web-based tool was created using the “DynNom” package and “shinyapps” in R. Statistical significance was set at *p* < 0.05.

## 3. Results

### 3.1. Baseline Characteristics

Initially, 161 biopsy-confirmed DN patients from the First Affiliated Hospital of Fujian Medical University were included. Sixteen patients were excluded due to concomitant primary or secondary kidney diseases, 2 patients were excluded due to a clinical diagnosis of Type 1 diabetes or steroid-induced diabetes, 8 patients were excluded due to reversible AKI during follow-up, and 18 patients were excluded due to incomplete data. Ultimately, 117 patients met the inclusion criteria (Figure 1). Patients were categorized into two groups, TLS− (*n* = 60) and TLS+ (*n* = 57), based on TLS assessment by immunohistochemical staining. The TLS+ group showed an elevated lymphocyte count (*p* = 0.026), elevated serum creatinine (*p* = 0.015), reduced baseline eGFR (*p* = 0.011), and increased urinary protein (*p* = 0.035) and UACR (*p* = 0.049). Pathological findings revealed more severe renal lesions in the TLS+ group, including higher DN glomerular classification, IFTA, and interstitial inflammation (all *p* < 0.05) (Table 1).

### 3.2. Risk Factors for More Mature TLS Formation in DN Patients

Comparison of baseline characteristics identified lymphocyte count, serum creatinine, baseline eGFR, urinary protein, UACR, DN glomerular classification, IFTA, and renal interstitial inflammation as potential factors influencing the formation of more mature TLS in DN patients. Binary logistic regression, accounting for multicollinearity, was used to adjust for confounding factors. Renal interstitial inflammation (*p* = 0.024) was identified as an independent risk factor for mature TLS formation in DN patients (Table 2).

### 3.3. Long-Term Renal Survival

**Figure 3:**
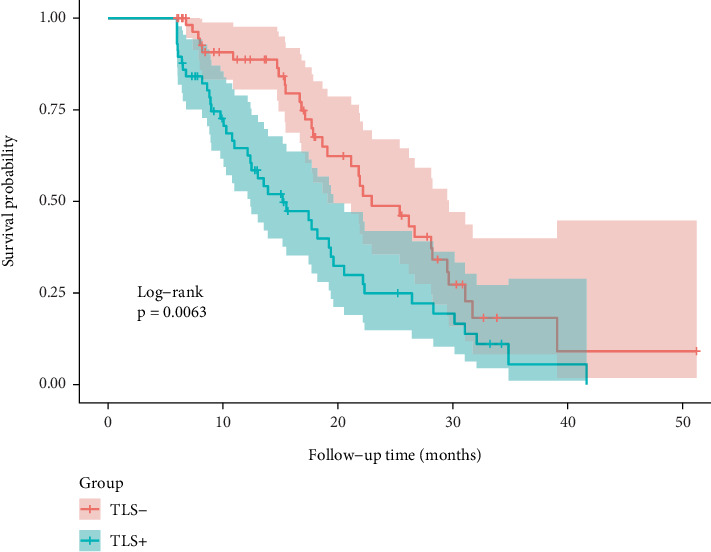
The Kaplan–Meier curve was used to compare the prognosis of patients with varying degrees of tertiary lymphoid structures across the entire cohort.

Renal endpoints were defined as an eGFR decrease > 25% in 1 year or an annual decline > 5 mL/min/1.73 m^2^. Kaplan–Meier survival analysis indicated renal function progression in 75 of 117 DN patients during a median follow-up period of 17 months. Renal function declined more frequently in the TLS+ group (75.4%, 43/57) than in the TLS− group (53.3%, 32/60). The TLS− group showed significantly better renal survival compared to the TLS+ group (*p* = 0.006, Figure 3).

### 3.4. Factors of Renal Endpoints

Univariate Cox regression (Table 3) identified serum creatinine, urinary protein, UACR, mature TLS, renal pathology classification, IFTA, and interstitial inflammation as factors influencing renal outcomes. Notably, in univariate analysis, systemic inflammatory markers including PLR (*p* = 0.163), NLR (*p* = 0.634), MLR (*p* = 0.744), SII (*p* = 0.305), SIRI (*p* = 0.795), RDW/ALB ratio (*p* = 0.090), and CRP (*p* = 0.777) all failed to reach statistical significance.

**Figure 4:**
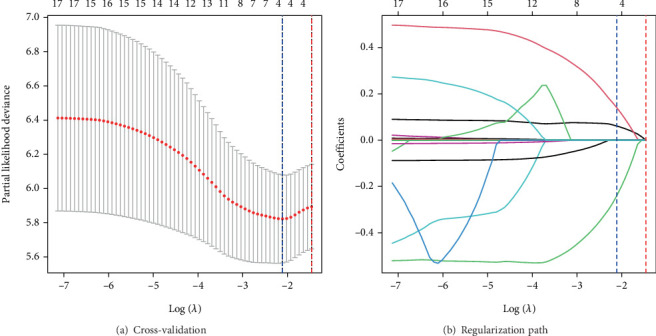
(a, b) LASSO-Cox regression variable selection.

Given the need to evaluate multiple potential confounding factors and considering that the number of variables exceeded the applicable range of traditional multivariate regression, LASSO-Cox regression was employed for feature selection. A total of 16 candidate variables were included in the analysis, comprising the seven significant variables mentioned above from univariate analysis, along with diabetes duration, glycated hemoglobin, and seven systemic inflammatory markers (PLR, NLR, MLR, SII, SIRI, RA, and CRP). Through 10-fold cross-validation, the optimal regularization parameter was determined as *λ* = 0.121 (log *λ* = −2.11). LASSO regression ultimately selected three variables with the highest predictive value from the 16 candidates: UACR, pathological classification, and TLS grouping (Figure 4). Final multivariate Cox regression analysis demonstrated that UACR (HR = 1.121, 95% CI: 1.038–1.210, *p* = 0.004), higher DN glomerular classification (HR = 1.511, 95% CI: 1.057–2.160, *p* = 0.024), and mature TLS (HR = 1.819, 95% CI: 1.144–2.893, *p* = 0.011) were independent predictors of renal outcomes in DN.

**Figure 5:**
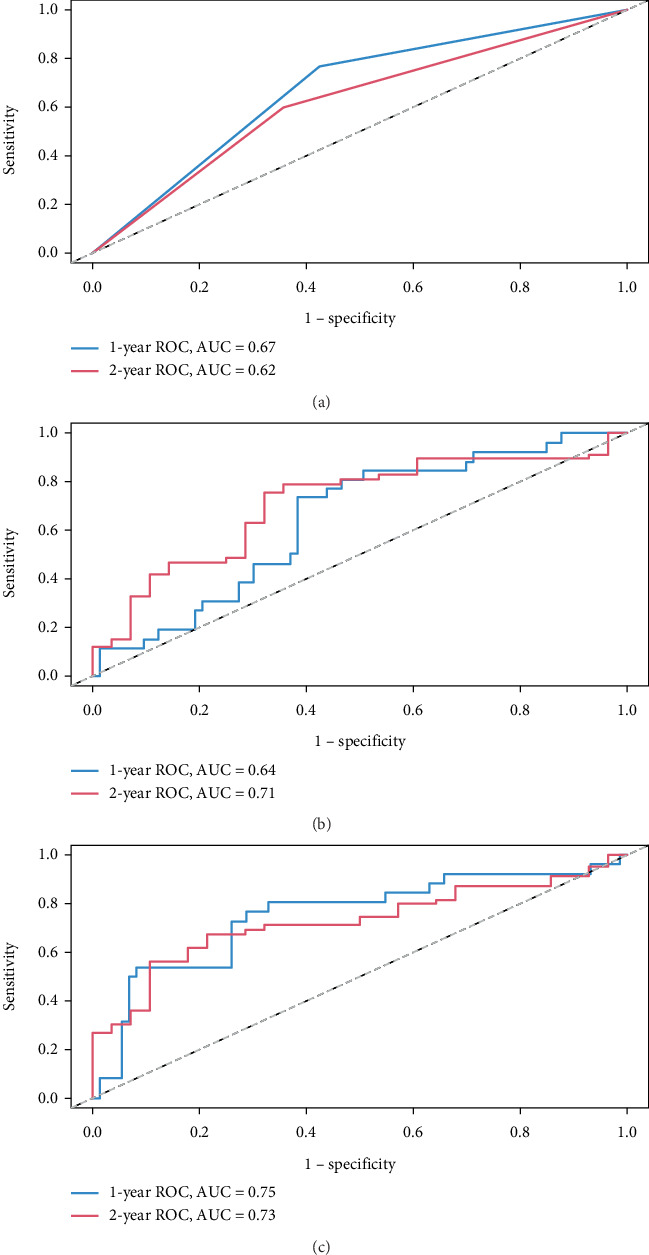
(a–c) Receiver operating characteristic curves for the three models.

**Figure 6:**
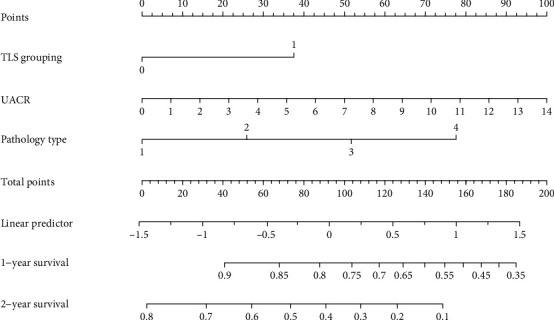
A nomogram was developed to predict renal function progression in DN patients with varying degrees of tertiary lymphoid structures.

Three predictive models were constructed based on key risk factors for renal progression. These models included a model based on TLS grouping alone (Model A), a model incorporating clinical indicators, including serum creatinine, urinary protein, and UACR (Model B), and a model based on significant indicators from the multivariate Cox regression analysis (UACR, renal pathology classification, and TLS grouping) (Model C). ROC curves assessed 1-year and 2-year renal survival rates (Figure 5). Model C demonstrated superior predictive performance, with a 1-year AUC of 0.75, compared to Model A (1-year AUC = 0.67) and Model B (1-year AUC = 0.64). Significant indicators from multivariate Cox regression were incorporated into a nomogram for predictive model development (Figure 6).

**Figure 7:**
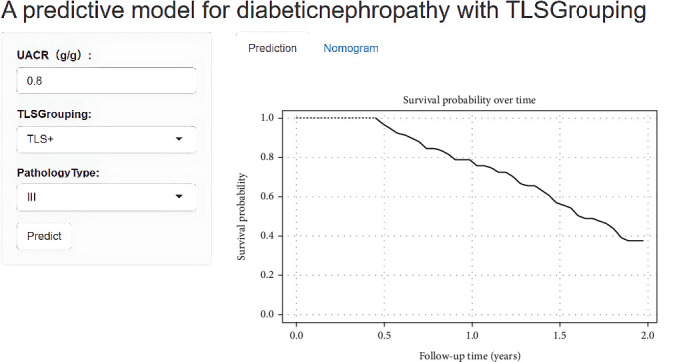
A screenshot of the web-based nomogram used to predict renal function progression in DN patients with varying degrees of tertiary lymphoid structures (https://fjmusnkmn.shinyapps.io/FJMUDN/).

An individualized web-based dynamic nomogram was created using the predictive model.  Figure 7 illustrates how the probability of renal disease progression can be estimated by summing variable scores, assisting clinicians in risk assessment. The predictive model is available online as a web application at https://fjmusnkmn.shinyapps.io/FJMUDN/.  Figure 7 demonstrates a clinical application example of the web-based model. Taking a DN patient with a UACR of 0.8 g/g, TLS+ grouping, and Type III pathological classification as an example, the model predicted 1-year and 2-year renal function progression probabilities of 22% and 63%, respectively. Based on this moderate-to-high risk prediction, intensive treatment and close follow-up strategies are recommended for this patient, including frequent monitoring (3–6 months' follow-up intervals), intensified renoprotective therapy (such as optimized ACEI/ARB use and strict blood pressure and glucose control), and enhanced lifestyle guidance and adherence management. This example illustrates the practical value of this predictive tool in guiding individualized clinical treatment decisions.

## 4. Discussion

Chronic inflammation, a hallmark of DN, links metabolic and hemodynamic abnormalities in diabetes to renal structural and functional damage [36]. DN is initiated by hyperglycemia-induced vascular dysfunction, and progression is driven by oxidative stress, inflammatory cell infiltration, and fibrosis. TLSs are organized lymphoid aggregates with specialized fibroblast networks that resemble the functional and structural characteristics of secondary lymphoid organs (SLOs), particularly lymph nodes. Unlike SLOs like lymph nodes, TLSs are less organized, lack a capsule, and exhibit unique features. Chronic inflammatory conditions, including cancer, infections, autoimmune diseases, and allograft rejection, can induce TLS formation in nonlymphoid organs [37–41]. Studies have shown that TLS development in minimally injured kidneys halts before reaching the germinal center stage, whereas TLS can fully mature in severely damaged kidneys. This finding highlights the correlation between TLS maturation and the severity of inflammation, renal injury, and renal dysfunction [42].

Our study demonstrates an association between renal TLS and disease progression in DN patients. Among 117 biopsy-confirmed DN patients, mature TLSs were associated with a poorer prognosis. Among the cohort, 53.3% (32/60) of patients in the TLS− group and 75.4% (43/57) of patients in the TLS+ group progressed to renal endpoints. Serum creatinine, urinary protein, UACR, more mature TLS, renal pathology classification, IFTA, and renal interstitial inflammation were significant in the univariate model. Considering the multifactorial complexity of DN prognosis, our study needed to simultaneously evaluate traditional clinical indicators (such as serum creatinine, urinary protein, and UACR), diabetes-related factors (diabetes duration, glycated hemoglobin), and systemic inflammatory markers (PLR, NLR, MLR, SII, SIRI, etc.). Therefore, LASSO-Cox regression was employed to assess the predictive value of TLS alongside other potential confounding factors. By incorporating 16 candidate variables into LASSO-Cox regression analysis, we found that only UACR, pathological classification, and TLS grouping were ultimately selected and retained, while all systemic inflammatory markers were excluded. This may be because routine blood parameters are susceptible to multiple confounding factors such as acute infections, medication treatments, and nutritional status. Additionally, systemic inflammatory markers lack organ specificity and cannot accurately reflect changes in the local inflammatory microenvironment of the kidney, whereas chronic kidney disease progression depends more on local pathological changes than on systemic inflammatory status. These results further confirm the unique advantage of local renal histological changes over systemic indicators in predicting DN prognosis. After adjusting for multicollinearity in multivariate Cox regression analysis, the results showed that more mature TLSs are an independent risk factor for renal function progression in DN patients. These results indicate that TLSs play a role in DN pathogenesis and may serve as predictors for disease progression.

In this study, renal interstitial inflammation was significant only in univariate Cox regression and identified as an independent risk factor for mature TLS formation in DN patients. This may be due to the assessment of renal interstitial inflammation, which includes both lymphocytes and monocytes, reflecting primarily short-term immune responses. However, in the long term, TLSs may better represent chronic immune responses and potentially play a more important role in prognosis. Age-related TLSs have been observed in the kidney, lung, and other organs, where they contribute significantly to chronic inflammatory diseases [42–47]. These findings highlight the critical role of TLS in regulating local immune responses, essential for designing strategies to mitigate chronic disease progression. Further studies are required to explore the mechanisms linking renal TLS with intrarenal inflammation and immunity.

In most patients, the first manifestation of DN is a slight increase in urinary albumin excretion [48]. High baseline albuminuria, regardless of diabetes severity, has been identified as a risk factor for poor renal outcomes. Controlled proteinuria results in similar rates of renal function decline between diabetic and nondiabetic CKD patients [49]. Another study found that patients with severely increased albuminuria (urinary albumin-to-creatinine ratio > 0.3 g/g) had an extremely high risk of renal function decline [50], which is consistent with our findings.

The RPS classification system, as a standardized tool for pathological assessment of DN, has had its prognostic predictive value validated in multiple studies. Early validation studies demonstrated that higher-grade lesions in the RPS classification were associated with increased risk of ESRD and dialysis requirements [51]. In our study, RPS pathological classification (HR = 1.511, 95% CI: 1.057–2.160, *p* = 0.024) was identified as an independent prognostic predictor, consistent with previous research findings, suggesting that the severity of glomerular lesions remains a key factor in prognostic prediction. However, the prognostic value of RPS classification remains somewhat controversial across different populations. A European cohort study found that in fully adjusted models, RPS classification showed no significant association with the initiation of renal replacement therapy [52]. Notably, the RPS classification primarily evaluates glomerular lesions and does not include specific assessment of renal inflammation, which is precisely why TLS assessment can provide important supplementary information.

Based on this understanding and after comparing predictive models, we selected a web-based dynamic model integrating TLS maturity, UACR, and renal pathology classification to assess renal progression risk over time. The web-based design offers ease of use, facilitating personalized risk predictions for patients and supporting clinical decision-making. This model can provide individualized risk stratification based on patients' specific pathological characteristics: high-risk patients can receive intensive treatment and close follow-up strategies, while patients with high UACR at diagnosis but low renal pathology classification and TLS maturity may have a low risk of renal progression, allowing for relatively conservative treatment strategies and extended follow-up intervals, thereby optimizing medical resource allocation. This individualized management approach based on risk stratification not only enhances the precision of clinical decision-making and the effectiveness of patient care but also provides a practical tool for precision medicine practice in DN.

This study has several limitations. First, including only biopsy-confirmed DN patients may introduce selection bias, limiting the generalizability of findings to patients with milder disease. Second, this single-center study with a limited sample size restricts broader applicability. Third, the median follow-up period of 17 months may be insufficient to fully assess long-term outcomes such as ESRD and mortality. Fourth, TLS assessment employed qualitative grading without formal interobserver reliability analysis, which may affect reproducibility. Therefore, large-scale, multicenter prospective studies with longer follow-up periods and standardized TLS scoring systems are needed to validate these findings.

## Figures and Tables

**Table 1:** Baseline characteristics of participants with DN according to tertiary lymphoid structures.

**Parameters**	**All (** **n** = 117**)**	**TLS− (** **n** = 60**)**	**TLS+ (** **n** = 57**)**	**p** ** value**
Clinical features				
Male sex (*n*)	87 (74.4%)	45 (75%)	42 (73.7%)	0.871
Age (years)	53.67 ± 11.70	52.57 ± 11.79	54.82 ± 11.61	0.299
Duration of diabetes (years)	10.00 (3.00, 10.00)	10.00 (3.00, 10.00)	7.00 (3.00, 10.00)	0.714
Hypertension (*n*)	102 (87.2%)	51 (85%)	51 (89.5%)	0.469
Cerebrovascular disease (*n*)	8 (6.8%)	3 (5%)	5 (8.8%)	0.483
Gout (*n*)	12 (10.3%)	4 (6.7%)	8 (14%)	0.189
BMI (kg/m^2^)	23.38 (21.64, 25.43)	23.44 (22.01, 25.66)	22.85 (21.63, 25.22)	0.345
Smoking (*n*)	37 (31.6%)	19 (31.7%)	18 (31.6%)	0.992
Hemoglobin (g/L)	111.68 ± 22.12	109.70 ± 24.36	113.77 ± 19.50	0.322
White blood cell (×10^9^/L)	6.79 (5.64, 8.14)	6.50 (5.05, 7.67)	7.29 (5.86, 8.39)	0.054
Neutrophil (×10^9^/L)	4.34 (3.53, 5.26)	4.25 (3.35, 4.80)	4.49 (3.77, 5.71)	0.115
Lymphocyte (×10^9^/L)	1.54 (1.20, 2.14)	1.39 (1.08, 1.91)	1.70 (1.32, 2.30)	0.026⁣^∗^
Monocyte (×10^9^/L)	0.41 (0.32, 0.49)	0.37 (0.30, 0.49)	0.42 (0.34, 0.52)	0.181
Platelet (×10^9^/L)	254.68 ± 76.81	251.35 ± 83.90	258.18 ± 69.13	0.633
RDW (%)	13.90 (13.10, 15.20)	13.90 (13.17, 15.60)	13.70 (13.00, 14.70)	0.278
CRP (mg/L)	2.32 (1.06, 5.20)	2.65 (1.18, 4.75)	1.99 (1.03, 5.20)	0.492
PLR	154.08 (116.84, 201.67)	158.80 (123.51, 216.07)	143.58 (116.43, 185.71)	0.159
NLR	2.70 (2.05, 3.47)	2.92 (2.06, 3.45)	2.41 (1.98, 3.55)	0.508
MLR	0.26 (0.19, 0.32)	0.28 (0.21, 0.32)	0.24 (0.18, 0.32)	0.253
SII	634.09 (484.61, 913.05)	668.85 (508.92, 909.28)	620.74 (463.97, 940.90)	0.532
SIRI	1.04 (0.77, 1.48)	1.01 (0.80, 1.47)	1.09 (0.77, 1.64)	0.967
RA	0.45 (0.37, 0.55)	0.46 (0.37, 0.55)	0.43 (0.37, 0.52)	0.693
HbA1c (%)	7.50 (6.40, 9.10)	7.85 (6.60, 9.43)	7.20 (6.40, 8.90)	0.135
Total bilirubin (*μ*mol/L)	5.20 (3.60, 7.50)	5.15 (3.80, 8.40)	5.30 (3.60, 7.50)	0.846
Serum albumin (g/L)	31.98 ± 7.04	32.37 ± 7.80	31.57 ± 6.17	0.540
Uric acid (*μ*mol/L)	369.00 (323.40, 430.70)	368.00 (315.73, 433.70)	370.00 (329.00, 430.00)	0.825
Urea nitrogen (mmol/L)	9.41 (6.86, 12.83)	8.73 (6.31, 12.32)	9.85 (7.15, 13.07)	0.386
Serum creatinine (*μ*mol/L)	129.00 (94.00, 170.00)	116.25 (87.00, 157.50)	152.50 (102.00, 198.00)	0.015⁣^∗^
eGFR (mL/min/1.73 m^2^)	50.87 (35.67, 75.61)	61.20 (43.94, 77.84)	41.39 (32.89, 65.58)	0.011⁣^∗^
Total cholesterol (mmol/L)	4.90 (4.18, 6.39)	4.86 (4.25, 6.47)	4.96 (3.96, 6.27)	0.748
Triglycerides (mmol/L)	1.75 (1.14, 2.36)	1.66 (1.09, 2.29)	1.80 (1.26, 2.45)	0.442
HDL-C (mmol/L)	1.06 (0.87, 1.31)	1.10 (0.88, 1.34)	1.02 (0.87, 1.30)	0.511
LDL-C (mmol/L)	3.16 (2.25, 4.37)	3.12 (2.30, 4.37)	3.17 (2.21, 4.48)	0.928
Calcium (mmol/L)	2.11 (2.00, 2.24)	2.13 (2.03, 2.24)	2.09 (1.99, 2.20)	0.330
Phosphorus (mmol/L)	1.24 (1.12, 1.40)	1.25 (1.12, 1.41)	1.22 (1.10, 1.39)	0.515
Potassium (mmol/L)	4.26 ± 0.60	4.20 ± 0.60	4.34 ± 0.59	0.207
Fibrinogen (g/L)	4.59 (3.59, 5.55)	4.36 (3.52, 5.26)	4.60 (3.76, 5.87)	0.216
Serum complement C3 (g/L)	0.86 (0.78, 1.03)	0.85 (0.79, 1.05)	0.87 (0.77, 1.00)	0.705
Serum complement C4 (g/L)	0.25 ± 0.09	0.24 ± 0.09	0.25 ± 0.08	0.488
Urinary protein (0/1+/2+/3+/4+)	6/8/26/54/23	4/5/16/27/8	2/3/10/27/15	0.035⁣^∗^
UACR (g/g)	2.82 (1.41, 4.95)	2.35 (1.11, 4.43)	3.24 (1.64, 5.68)	0.049⁣^∗^
Renal pathology				
Glomerular classification (I/II/III/IV)	3/25/65/24	1/19/34/6	2/6/31/18	0.001⁣^∗^
IFTA percentage (%)	40.00 (20.00, 50.00)	30.00 (13.75, 40.00)	40.00 (30.00, 60.00)	0.004⁣^∗^
IFTA score (0/1/2/3)	0/38/39/40	0/26/20/14	0/12/19/26	0.003⁣^∗^
Interstitial inflammation (%)	30.00 (15.00, 50.00)	20.00 (10.00, 30.00)	40.00 (20.00, 50.00)	< 0.001⁣^∗∗^
Kimmelstiel–Wilson nodules (*n*)	78 (66.7%)	36 (60%)	42 (73.7%)	0.117
Hyaline arteriolosclerosis (*n*)	110 (94%)	57 (95%)	53 (93%)	0.712
CD68 deposition (−/focal+/abundant+)	30/78/9	16/40/4	14/38/5	0.695
Medications				
ACEI/ARB (*n*)	77 (65.8%)	41 (68.3%)	36 (63.2%)	0.555
Lipid-lowering therapy (*n*)	40 (34.2%)	19 (31.7%)	21 (36.8%)	0.555

*Note:* Data are presented as the mean ± standard, median (quartile range) or percentages.

Abbreviations: ACEI/ARB, angiotensin-converting enzyme inhibitor/angiotensin II receptor blocker; CRP, C-reactive protein; eGFR, estimated glomerular filtration rate; HDL-C, high-density lipoprotein cholesterol; IFTA, interstitial fibrosis/tubular atrophy; LDL-C, low-density lipoprotein cholesterol; MLR, monocyte-to-lymphocyte ratio; NLR, neutrophil-to-lymphocyte ratio; PLR, platelet-to-lymphocyte ratio; RA, red cell distribution width/serum albumin ratio; RDW, red cell distribution width; SII, systemic immune-inflammation index; SIRI, systemic inflammation response index; UACR, urinary albumin-to-creatinine ratio.

⁣^∗^*p* < 0.05. ⁣^∗∗^*p* < 0.001.

**Table 2:** Multifactorial analysis of DN patients with tertiary lymphoid structures (logistic).

**Parameters**	**OR (95% CI)**	**p** ** value**
Clinical features		
Lymphocyte	1.620 (0.907, 2.893)	0.103
Serum creatinine	1.002 (0.991, 1.013)	0.782
eGFR	0.995 (0.966, 1.025)	0.759
Urinary protein	1.247 (0.723, 2.151)	0.427
UACR	1.062 (0.868, 1.299)	0.561
Renal pathology		
Glomerular classification	1.707 (0.861, 3.386)	0.126
IFTA percentage	0.968 (0.926, 1.012)	0.156
Interstitial inflammation	1.052 (1.007, 1.099)	0.024⁣^∗^

Abbreviations: 95% CI, 95% confidence interval; eGFR, estimated glomerular filtration rate; IFTA, interstitial fibrosis/tubular atrophy; UACR, urinary albumin-to-creatinine ratio.

⁣^∗^*p* < 0.05.

**Table 3:** Risk factors for renal endpoint determined by univariate/multivariate Cox hazard analysis in DN.

**Parameters**	**Univariate**		**Multivariate**	
	**HR (95% CI)**	**p** ** value**	**HR (95% CI)**	**p** ** value**
Clinical features				
Male sex	0.609 (0.363, 1.020)	0.060		
Age	0.993 (0.976, 1.011)	0.469		
Duration of diabetes	0.994 (0.960, 1.030)	0.744		
Hypertension	0.903 (0.412, 1.978)	0.799		
Cerebrovascular disease	1.477 (0.529, 4.123)	0.457		
Gout	0.656 (0.313, 1.376)	0.265		
BMI	0.949 (0.874, 1.031)	0.214		
Smoking	0.644 (0.385, 1.076)	0.093		
Hemoglobin	0.989 (0.979, 1.000)	0.053		
White blood cell	0.973 (0.856, 1.106)	0.677		
Neutrophil	1.012 (0.856, 1.197)	0.885		
Lymphocyte	0.884 (0.635, 1.230)	0.465		
Monocyte	0.234 (0.040, 1.363)	0.106		
Platelet	1.003 (1.000, 1.006)	0.089		
RDW	1.153 (0.987, 1.346)	0.072		
CRP	0.996 (0.967, 1.025)	0.777		
PLR	1.002 (0.999, 1.004)	0.163		
NLR	1.031 (0.909, 1.170)	0.634		
MLR	0.743 (0.126, 4.405)	0.744		
SII	1.000 (1.000, 1.001)	0.305		
SIRI	0.966 (0.741, 1.258)	0.795		
RA	4.366 (0.794, 24.007)	0.090		
HbA1c	0.935 (0.834, 1.048)	0.247		
Total bilirubin	0.980 (0.927, 1.036)	0.475		
Serum albumin	0.974 (0.942, 1.008)	0.129		
Uric acid	0.999 (0.997, 1.002)	0.590		
Urea nitrogen	1.018 (0.976, 1.062)	0.399		
Serum creatinine	1.004 (1.000, 1.007)	0.027⁣^∗^		
eGFR	0.993 (0.984, 1.002)	0.118		
Total cholesterol	1.056 (0.981, 1.136)	0.144		
Triglycerides	1.087 (0.955, 1.238)	0.205		
HDL-C	0.865 (0.507, 1.476)	0.595		
LDL-C	1.066 (0.954, 1.191)	0.256		
Calcium	0.558 (0.139, 2.245)	0.412		
Phosphorus	1.690 (0.788, 3.624)	0.178		
Potassium	1.150 (0.749, 1.765)	0.523		
Fibrinogen	1.115 (0.948, 1.311)	0.187		
Serum complement C3	0.707 (0.219, 2.283)	0.562		
Serum complement C4	0.323 (0.022, 4.813)	0.412		
Urinary protein	1.415 (1.072, 1.867)	0.014⁣^∗^		
UACR	1.140 (1.056, 1.229)	0.001⁣^∗^	1.121 (1.038, 1.210)	0.004⁣^∗^
Renal pathology				
TLS grouping	1.881 (1.188, 2.981)	0.007⁣^∗^	1.819 (1.144, 2.893)	0.011⁣^∗^
Glomerular classification	1.662 (1.169, 2.364)	0.005⁣^∗^	1.511 (1.057, 2.160)	0.024⁣^∗^
IFTA percentage	1.013 (1.002, 1.024)	0.017⁣^∗^		
IFTA score	1.320 (0.987, 1.765)	0.062		
Interstitial inflammation	1.015 (1.004, 1.026)	0.008⁣^∗^		
Kimmelstiel–Wilson nodules	1.601 (0.940, 2.728)	0.083		
Hyaline arteriolosclerosis	1.820 (0.572, 5.789)	0.310		
Renal CD68 deposition	1.061 (0.703, 1.602)	0.778		
Medications				
ACEI/ARB	0.728 (0.451, 1.176)	0.195		
Lipid-lowering therapy	1.232 (0.755, 2.011)	0.404		

Abbreviations: 95% CI, 95% confidence interval; ACEI/ARB, angiotensin-converting enzyme inhibitor/angiotensin II receptor blocker; CRP, C-reactive protein; eGFR, estimated glomerular filtration rate; HDL-C, high-density lipoprotein cholesterol; HR, hazard ratio; IFTA, interstitial fibrosis/tubular atrophy; LDL-C, low-density lipoprotein cholesterol; MLR, monocyte-to-lymphocyte ratio; NLR, neutrophil-to-lymphocyte ratio; PLR, platelet-to-lymphocyte ratio; RA, red cell distribution width/serum albumin ratio; RDW, red cell distribution width; SII, systemic immune-inflammation index; SIRI, systemic inflammation response index; TLS, tertiary lymphoid structure; UACR, urinary albumin-to-creatinine ratio.

⁣^∗^*p* < 0.05.

## Data Availability

The original data utilized in this study can be obtained from the corresponding authors upon reasonable request.
